# Optimizing the physical activity intervention for older adults with mild cognitive impairment: a factorial randomized trial

**DOI:** 10.3389/fspor.2024.1383325

**Published:** 2024-05-07

**Authors:** Zhanfang Shao, Jundan Huang, Hui Feng, Mingyue Hu

**Affiliations:** ^1^ Department of Nursing, Peking Union Medical College Hospital, Beijing, China; ^2^ Xiangya School of Nursing, Central South University, Changsha, Hunan, China

**Keywords:** physical activity, mild cognitive decline, old adults, factorial randomized trial, multiphase optimization strategy

## Abstract

**Background:**

Physical activity (PA) intervention is one of the most effective interventions to promote cognitive function of older adults with mild cognitive impairment (MCI). However, the level of PA remains low. Based on the two core interventions (X-CircuiT and health education), this study aimed to examine the effect of three implementation strategies (viz., role modeling, goal-setting, and reminding) on the PA level among older adults with MCI using the multiphase optimization strategy (MOST).

**Methods:**

Participants were randomized into one of eight conditions in a factorial design involving three factors with two levels: (i) role modeling (on vs. off); (ii) goal-setting (on vs. off); and (iii) reminding (on vs. off). The primary outcome was PA level at 12 weeks. The secondary outcomes were cognitive function, self-efficacy, and cost-effectiveness at 12 weeks. The intention-to-treat (ITT) analysis was performed as the main analysis and the per-protocol (PP) analysis as the sensitivity analysis.

**Results:**

A total of 107 participants were included and randomly assigned into three groups, each receiving different implementation strategies. The results of the multivariate regression analysis showed that the three implementation strategies, namely, reminding (*B* = 0.31, *p* < 0.01), role modeling (*B* = 0.21, *p* < 0.01), and goal-setting (*B* = 0.19, *p* < 0.01), could significantly improve PA level. Specifically, it was found that role modeling (*B* = 0.68, *p* = 0.03) could significantly improve cognitive function. There were no significant interactions among the three implementation strategies. Role modeling was the most cost-effective strategy, costing 93.41 RMB for one unit of PA.

**Conclusions:**

Role modeling was likely to be the best implementation strategy. The value-based and cost-effective PA intervention package could include the core intervention (X-CircuiT and health education) and implementation strategy (role modeling).

**Clinical Trial Registration:**

https://www.chictr.org.cn, The study was retrospectively registered on 30 June 2022 (ChiCTR2200061693).

## 1 Background

Dementia is the seventh leading cause of all diseases and a prominent contributor to disability and dependency among older adults worldwide (1). As of 2021, 55 million people are living with dementia globally, a number which is projected to increase to 78 million by 2030 and 139 million by 2050 (2). Fortunately, delaying the onset of dementia by 5 years could potentially reduce the prevalence of dementia worldwide by half (3). Mild cognitive impairment (MCI) is an intermediate stage between normal cognition and dementia, with a progression rate of 46% to dementia within 3 years, compared with only 3% of the age-matched population (4). Therefore, MCI is an important stage in preventing the progression of dementia.

Physical inactivity is an important modifiable risk factor in the development of dementia (5). Many studies have shown the effect of physical activity (PA) on the cognitive function of older adults. A meta-analysis comprising 15 prospective studies, involving a total of 33,816 participants, has shown that engaging in PA, regardless of its intensity, could delay the development of cognitive decline (6). An umbrella review with 27 systematic reviews and 28,205 participants showed that PA was significantly associated with cognitive and noncognitive outcomes among individuals with MCI (7). The Xiangya Hospital circuit training (X-CircuiT), a multicomponent exercise program developed by the cardiac rehabilitation team based on current multinational guidelines, could take into account the PA preferences of older adults with pre-frailty and effectively reverse pre-frailty (8, 9). The potential mechanisms by which PA promotes cognitive function include the release of growth factors, inflammatory cytokines, neuroplasticity across the brain, the production of lactate, increased mitochondrial biogenesis, and enhanced activity of the antioxidant enzyme (10, 11).

Despite significant cognitive benefits from PA, the PA level among older adults is not ideal. One study including 129,400 older adults in the National Health Interview Survey found that only 8.6% of older adults met the recommendation for sufficient PA (12). A pilot study found that adults with MCI spent about only 89 min in light PA, accumulated about an average of 863 (standard deviation of 699) steps per day in average, and spent 87.2% of the accelerometer wear time in sedentary behavior (13).

Several implementation strategies have been identified to increase the PA level in older adults with cognitive decline, but there is no consistent approach to the type of strategies being used. A recent systematic review has demonstrated that several implementation strategies, such as role modeling, goal-setting, and reminding, have proven effective in improving the PA level of participants (14). One RCT utilized text messages to remind participants aged 55–70 years to exercise and found that those who received reminders exercised 1.21 times as much as the control group (15). One recent study found that a specifically designed home-based physical training and activity promotion program could improve physical capacity (16). Meanwhile, many behavior change techniques involving skills, knowledge, and motivation are used to promote PA in older adults in future information and communication technology interventions (17). The social cognitive theory (SCT) provides a theoretical framework for understanding the underlying mechanisms of how implementation strategies enhance PA. These implementation strategies could optimize facilitators and mitigate barriers from personal, environmental, and behavioral perspectives (18), ultimately leading to improved PA levels. However, the optimal implementation strategy or combination of strategies to enhance intervention effectiveness remains unclear.

Therefore, based on the SCT, this study aims to evaluate the effectiveness of three implementation strategies using the multiphase optimization strategy (MOST) study design.

## 2 Methods

### 2.1 Conceptual model

**Figure 1:**
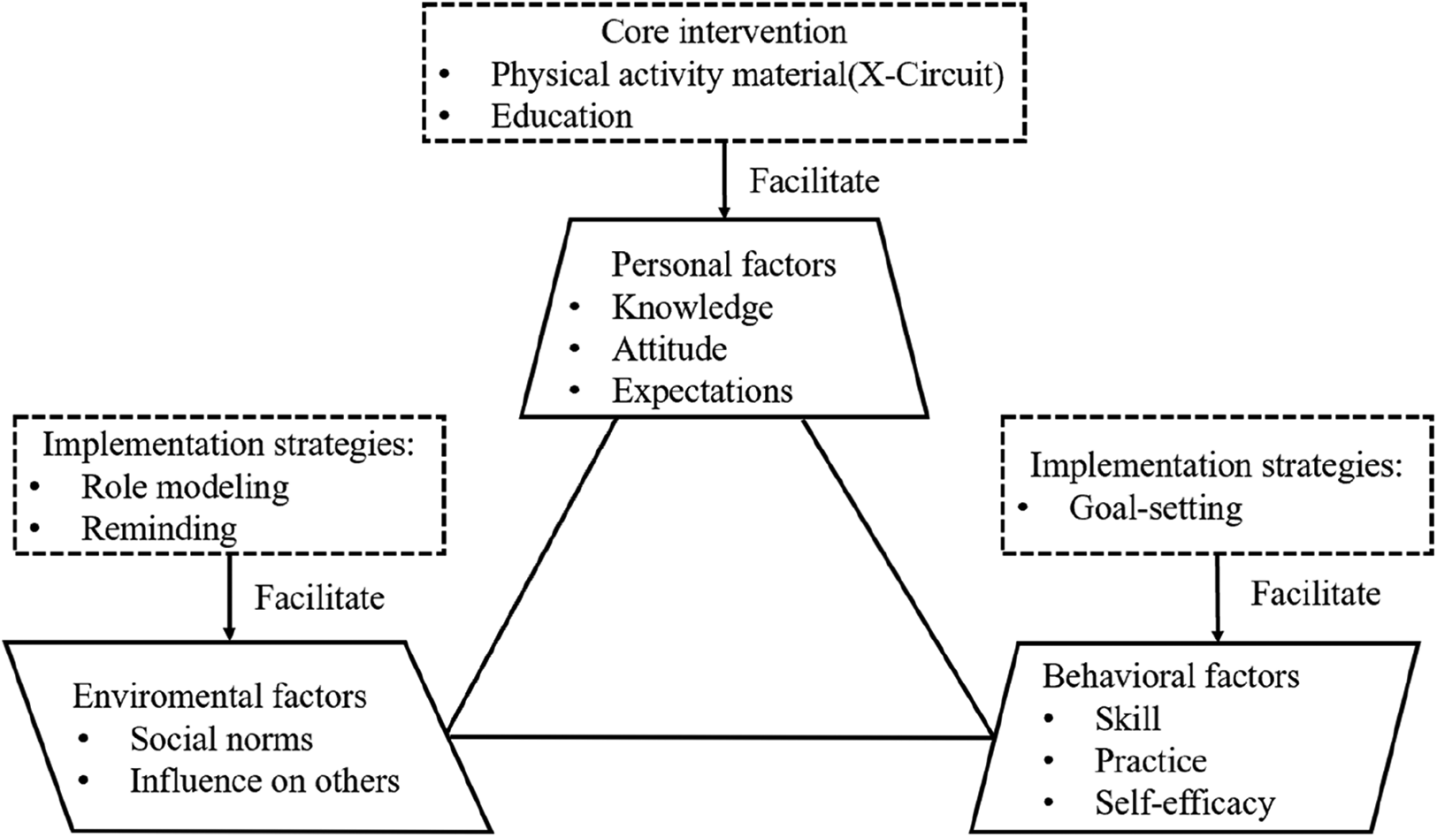
The hypothesized conceptual model is composed of core intervention and implementation strategies based on SCT.

According to the SCT and previous studies, a total of two core intervention components (PA material and PA education) and three implementation strategies (role modeling, reminding, and goal-setting) (14, 19) were selected. The hypothesized conceptual model based on SCT is shown in Figure 1.

#### 2.1.1 Core intervention

X-CircuiT was used as a PA material (see Supplementary Figure S1). This consisted of five distinct stages: warm-up (4.5 min), aerobic exercise (6.5 min), acupoint patting (6.0 min), resistance training (15 min), and flexibility training (14 min).

The education program (20, 21) consisted of three seminars on topics relevant to PA and cognitive impairment (“understanding cognitive decline,” “risk factors of cognitive decline,” and “physical activity as an effective intervention and safety security”). The educational sessions were offered for all participants in the community once a month.

#### 2.1.2 Implementation strategies

##### 2.1.2.1 Role modeling (face-to-face strategy)

The group members were guided by a respected role model who assumed the responsibility of leading the participants to engage in PA together and coordinate with community managers for administrative tasks (22). The participants were provided with in-person role modeling sessions once a week for 12 weeks in the community exercise laboratory.

##### 2.1.2.2 Reminding (internet-delivered strategy)

Older adults in this group received text messages that reminded them to perform PA. Reminding could increase the awareness of participants to perform PA (14). The frequency, order, repetition, timing, and direction of text messages were determined based on the participants' preferences and the WHO guidelines (23). During the first month, the participants received text messages once a week (four text messages). In the next 2 months, they received text messages twice a week (four text messages).

##### 2.1.2.3 Goal-setting (Internet-delivered strategy)

Older adults in this group received a weekly task and self-monitored their PA. Goal-setting enabled the participants to assess their current PA level and monitor their performance in comparison to goals (24). The participants in this group could set personalized goals based on their physical conditions. However, they were asked to record goal attainment.

### 2.2 Study design

The study was a 2 × 2 × 2 factorial, single-blinding, randomized controlled trial conducted from August 2021 to March 2022. This experiment consisted of three implementation strategies (viz., role modeling, reminding, and goal-setting), each compromising active (on) condition and control (off) condition, resulting in a total of eight experimental conditions (see Table 1). A summary description of the protocol is available at https://www.chictr.org.cn (ChiCTR2200061693). The study followed the Consolidated Standards of Reporting Trial (CONSORT) 2010 reporting guideline.

**Table 1:** Experimental groupings and implementation strategies.

Experimental groupings	Core interventions	Implementation strategies		
		Role modeling	Reminding	Goal-setting
1	+	+	+	+
2	+	+	+	−
3	+	+	−	+
4	+	+	−	−
5	+	−	+	+
6	+	−	+	−
7	+	−	−	+
8	+	−	−	−

+, on; −, off.

### 2.3 Participants

Participants were recruited from July 2021 to August 2021 in four communities from Changsha City, Hunan Province, China. The non-probability and convenience sampling method was used to recruit study participants.

The inclusion criteria were as follows: (1) older adults aged ≥60 years who were able to walk to community health service centers; (2) older adults who had been diagnosed with MCI according to Peterson's criteria [Montreal Cognitive Assessment (MoCA) score <26 for those with 12 years or more of education and <25 for those with less than 12 years of education; activity of daily living scale ≤23; no clinical diagnosis of dementia] (21, 25); (3) older adults with current PA level lower than that recommended by the Word Health Organization (WHO) guidelines (26) (at least 150–300 min of moderate-intensity aerobic PA; at least 75–150 min of vigorous-intensity aerobic PA; or an equivalent combination of moderate- and vigorous-intensity activity throughout 1 week); and (4) older adults who were physically normal (the six-item Katz Activities of Daily Living Scale should all be evaluated as “independent”).

The exclusion criteria were as follows: (1) older adults who were diagnosed with depression and (2) those with potential PA risk (who answered “yes” to any item of the Physical Activity Readiness Questionnaire).

### 2.4 Outcomes

#### 2.4.1 Baseline characteristics

Participants’ demographic information, including general characteristics, lifestyles, and comorbid conditions, was collected at baseline. The general characteristics included age, gender, education, and occupation. The lifestyles included smoking and drinking. The comorbid conditions included hypertension, diabetes, and stroke.

#### 2.4.2 Primary outcome

The primary outcome was PA level, which was measured using the Physical Activity Scale for the Elderly (PASE). This is a 10-item scale designed to assess the PA level among older adults (27). The total PASE score was computed by multiplying the time spent in each activity (hours per week) and participation (i.e., yes/no) in an activity, by empirically derived item weights, and then by summing the overall activities. The total PASE score ranges from 0 to 400, with a higher score indicating a higher PA level. It has demonstrated acceptable reliability (with a test–retest coefficient of 0.90) and validity rates in Chinese older adults (28).

#### 2.4.3 Secondary outcomes

##### 2.4.3.1 Cognitive function

Cognitive function was assessed by the Subjective Cognitive Decline Questionnaire (SCD-Q9) and the MoCA. SCD-Q9 (29) consists of nine items designed for the early identification of older adults with subject cognitive decline. The total score ranges from 0 to 21, with a higher score indicating a higher likelihood of cognitive impairment. It has demonstrated acceptable reliability (with a Cronbach's *α* of 0.90) and validity rates in Chinese older adults (30, 31). MoCA (32) is used to assess several cognitive domains including memory, visual ability, attention, execution, calculation, and orientation. The total score ranges from 0 to 30, with a higher score indicating better performance. It has demonstrated acceptable reliability (with a Cronbach's *α* of 0.82) and validity rates in Chinese elderly subjects (33).

##### 2.4.3.2 Self-efficacy

Self-efficacy was measured by the Self-Efficacy for Exercise Scale (SEE) (34). This is a nine-item scale designed to evaluate the perception of confidence to engage in regular exercise. The total score ranges from 0 to 90, with a higher score representing better exercise self-efficacy. It has demonstrated acceptable reliability (with a Cronbach's *α* of 0.75) and validity rates in Chinese older adults (35).

##### 2.4.3.3 Cost-effectiveness

The cost-effectiveness analysis was evaluated using the assessed cost-effectiveness ratio (CER), which is calculated as follows: CER = Cost (C) / Effectiveness (E) = total cost / increased PA. This ratio indicates the cost of increasing each unit of PA. The total cost was calculated from the perspective of community managers. Older adults have free access to X-CircuiT and implementation strategies due to several aging policies in China. The cost primarily comprised labor fees for individuals working in the communities, education fees for those delivering lectures, and other unforeseen expenses fees (see Supplementary Table S1).

### 2.5 Sample size

As each implementation strategy is allocated to half of the participants, the sample size required to detect the main effects in a full factorial following MOST design does not depend on the number of implementation strategies evaluated but rather on the smallest clinically important difference between the presence and absence of an implementation strategy (36). According to a previous study, we assumed an effect size of 0.50 for the main effects of each implementation strategy (37). We also assumed that when the outcome was used as the baseline variable, the correlation coefficient with the outcome was 0.50. With a two-sided significance level of 0.05 and a power of 80%, a total sample size of 97 was needed. Considering a 10% attrition rate, we decided to include 107 participants. The sample size was calculated using the “MOST” package from R Version 4.1.2.

### 2.6 Randomization

The participants were randomly assigned to one of the eight experimental conditions using computer-generated random numbers (MOST package “Random Assignment Generator”). The computer-generated randomization was performed by a statistician who was involved in the study design and conduction. Allocation concealment was implemented by sealed opaque envelopes distributed by the statistician.

### 2.7 Blinding

The outcome assessors maintained blinding throughout the intervention process, while the inability to blind the study personnel and participants was attributed to the unique nature of the PA intervention.

### 2.8 Safety measurement

The security measures were conducted to ensure the safety of older adults. First, the PAR-Q assessment was performed at baseline. All PAR-Q questions must be answered as “no” when including participants. Second, participants were asked to stop PA if any item of PAR-Q questions was answered as “yes” during the intervention. Adverse events, which are defined as injuries caused by the intervention, were recorded, and medical care was offered by the general practitioners.

### 2.9 Statistical analysis

In the descriptive analysis, the categorical variables were described by frequency with proportion. The continuous variables were described by mean ± standard deviation (mean ± SD) for normal distribution data and median and interquartile range (IQR) for non-normal distribution data. The normally distributed quantitative data were analyzed by analysis of variance (ANOVA). Otherwise, the non-parametric tests were used. A chi-squared test was performed for the categorical variables. The potential confounders were considered in subsequent models if they exhibited a significant correlation with the outcomes (*p* < 0.05). Normality was evaluated using the Shapiro–Wilk test. Histogram and log transformation were applied when deemed necessary. Random missing data were addressed using multiple imputations.

The outcome focused on the differences in PA, cognitive function, self-efficacy, and cost-effectiveness at 12 weeks between the intervention and control groups with a linear regression analysis conducted while adjusting for potential confounders. Notably, the effect coding (each coded −1 = off condition; +1 = on condition) of the implementation strategies was used to test the main and interactive effects instead of dummy coding (0, 1) (38). Intention-to-treat (ITT) analysis was used as the main analysis, and per-protocol (PP) analysis was used as the sensitivity analysis. For the treatment of missing data, the outcome indicators were still evaluated in the dropout population. For the population lost to follow-up, baseline data were used in place of outcome data. IBM Statistic SPSS 25.0 and R 4.1.2 software were used for statistical analysis.

## 3 Results

### 3.1 Participants

**Figure 2:**
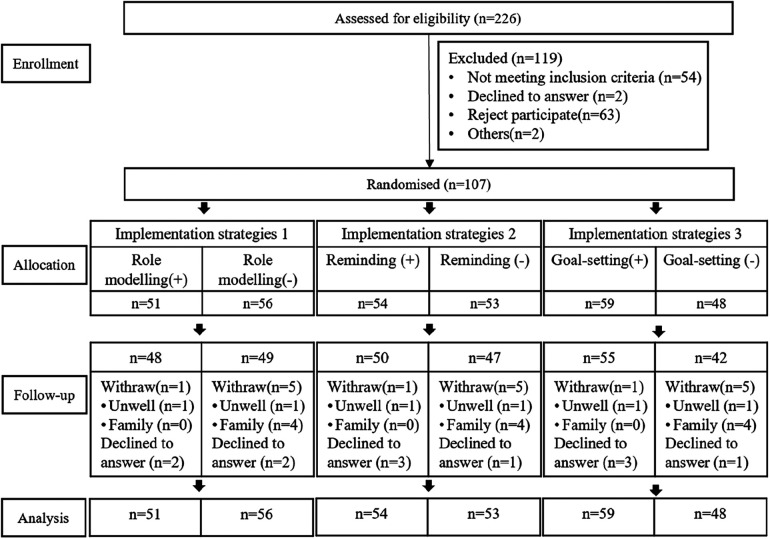
CONSORT diagram.

Among the 226 potential individuals screened, 107 were eligible and agreed to participate in the study. The intervention subjects were randomly assigned as shown in Figure 2. The median age of participants was 67.0 years. A total of 23 (23.4%) participants were male. At baseline, the intervention and control groups were significantly different in age, gender, education, occupation, and smoking (*p* < 0.05). The baseline information about the study participants has been presented in Table 2.

**Table 2:** Baseline information on study participants (*n* = 107).

Characteristics	Variable	*n* (%)/median [quartile]	Reminding			Role modeling			Goal-setting		
			+	−	*p*	+	−	*p*	+	−	*p*
Age	–	67.0 [62.0, 73.0]	67.5 [63.0, 75.5]	65.0 [61.0, 73.0]	0.09	72.0 [63.0, 78]	64.5 [61.0, 69.0]	**0**.**01**	67.0 [62.0, 74.0]	69.0 [61.3, 72.8]	0.87
Gender	Male	25 (23.4)	16 (29.6)	9 (17.0)	0.12	19 (37.3)	6 (10.7)	**0**.**01**	20 (33.9)	5 (10.4)	**0**.**04**
	Female	82 (76.6)	38 (70.4)	44 (83.0)	** **	32 (62.7)	50 (89.3)		39 (66.1)	43 (89.6)	
Education	Primary school and below	28 (27.2)	9 (16.7)	19 (35.8)	0.08	7 (13.8)	21 (37.5)	**0**.**02**	7 (11.9)	21 (43.8)	**0**.**00**
	Middle school	31 (29.0)	18 (33.3)	13 (24.5)	** **	19 (37.3)	12 (21.4)		24 (40.7)	7 (14.6)	
	High school and above	48 (44.8)	27 (50.0)	21 (39.6)	** **	25 (49.0)	23 (41.1)		28 (47.5)	20 (41.7)	
Occupation	Agricultural	49 (45.8)	19 (35.2)	30 (56.6)	**0**.**03**	13 (25.5)	36 (64.3)	**0**.**00**	20 (33.9)	29 (60.4)	**0**.**01**
	Non-agricultural	58 (54.2)	35 (64.8)	23 (43.4)	** **	38 (74.5)	20 (35.7)		39 (66.1)	19 (39.6)	
Smoking	Never	87 (81.3)	49 (90.7)	38 (71.7)	0.01	47 (92.2)	40 (71.4)	**0**.**01**	51 (86.4)	36 (75.0)	0.13
	Current or former	20 (18.7)	5 (9.4)	15 (28.3)	** **	4 (7.8)	16 (28.6)		8 (13.6)	12 (25.0)	
Drinking	Never	81 (75.7)	44 (81.5)	37 (69.8)	0.16	40 (78.4)	41 (73.2)	0.53	44 (74.6)	37 (77.1)	0.76
	Drinking/quit drinking	26 (24.3)	10 (18.5)	16 (30.2)		11 (21.6)	15 (26.8)		15 (25.4)	11 (22.9)	
Hypertension	No	76 (71.0)	39 (72.2)	37 (69.8)	0.78	37 (72.5)	39 (69.6)	0.74	44 (74.6)	32 (66.7)	0.37
	Yes	31 (29.0)	15 (27.8)	16 (30.2)		14 (27.5)	17 (30.4)		15 (25.4)	16 (33.3)	
Diabetes	No	82 (76.6)	44 (81.5)	38 (71.7)	0.23	38 (74.5)	44 (78.6)	0.62	45 (76.3)	37 (77.1)	0.92
	Yes	25 (23.4)	10 (18.5)	15 (28.3)		13 (25.5)	12 (21.4)		14 (23.7)	11 (22.9)	
Stroke	No	99 (92.5)	49 (90.7)	50 (94.3)	0.73	45 (88.2)	54 (96.4)	0.21	54 (91.5)	45 (93.8)	0.95
	Yes	8 (7.5)	5 (9.3)	3 (5.7)		6 (11.8)	2 (3.6)		5 (8.5)	3 (6.3)	
PASE^a^	–	58.6 [50.7, 87.1]	55.4 [50.7, 77.9]	64.3 [46.5, 106.2]	0.20	69.3 [55.0, 95.8]	58.6 [35.0, 79.6]	0.07	58.6 [52.1, 87.5]	59.8 [26.1, 84.5]	0.57
SEE^b^	–	66.0 [48.0, 77.0]	66.0 [55.5, 73.3]	63.0 [41.0, 77.5]	0.45	66.0 [37.0, 74.0]	64.5 [48.0, 77.8]	0.80	66.0 [51.0, 77.0]	60.0 [47.3, 76.8]	0.35
SCD^c^	–	6.0 [4.0, 7.0]	6.3 [4.0, 7.1]	6.0 [3.8, 6.5]	0.21	6.5 [4.5, 7.5]	6.0 [3.5, 6.5]	0.36	6.5 [5.5, 7.5]	6.0 [3.5, 6.5]	0.06
MoCA^d^	–		18.0 [13.75, 23.0]	20.0 [15.0, 23.0]	0.47	20.0 [15.0, 23.0]	17.5 [15.0, 22.0]	0.40	18.0 [14.0, 23.0]	19.0 [15.0, 22.8]	0.99

+, on; −, off.

Significant *p*-values are in bold.

^a^ PASE, Physical Activity Scale for the Elderly.

^b^ SEE, Self-Efficacy for Exercise Scale.

^c^ SCD, subjective cognitive decline.

^d^ MoCA, Montreal Cognitive Assessment.

### 3.2 PA level

The results showed that reminding (*B* = 0.23, *p* < 0.01), role modeling (*B* = 0.40, *p* < 0.01), and goal-setting (*B* = 0.32, *p* < 0.01) significantly increased PA at 12 weeks in all three implementation strategies. After adjusting for age, gender, education, occupation, smoking, and baseline PA, reminding (*B* = 0.21, *p *< 0.01), role modeling (*B* = 0.31, *p* < 0.01), and goal-setting (*B* = 0.19, *p* < 0.01) significantly increased PA at 12 weeks. In the interaction analysis, the antagonistic effect was not significant after adjustment. There was a synergistic effect among the three implementation strategies (*B* = 0.32, *p* < 0.01) (see Table 3).

**Table 3:** The effects of three implementation strategies on outcomes (ITT^a^ analysis).

Implementation strategies	PASE^b^^,^*				ESS^c^				SCD^d^				MoCA^e^			
	No adjusted		Adjusted^f^		No adjusted		Adjusted^g^		No adjusted		Adjusted^h^		No adjusted		Adjusted^i^	
	B	*P*	B	*p*	B	*p*	B	*p*	B	*p*	B	*P*	B	*p*	B	*p*
Reminding	0.23	**<0.01**	0.21	**<0.01**	3.62	0.06	0.61	**<0.01**	−0.35	0.09	−0.39	**0**.**02**	−0.44	0.41	−0.08	0.78
Role modeling	0.40	**<0.01**	0.31	**<0.01**	7.47	**<0.01**	0.64	**<0.01**	−0.54	**0**.**01**	−0.53	**0**.**01**	0.90	0.09	0.68	**0**.**03**
Goal-setting	0.32	**<0.01**	0.19	**<0.01**	4.39	**0**.**02**	0.60	**<0.01**	−0.06	0.76	−0.09	0.64	−0.20	0.71	−0.17	0.57
Reminding × role modeling	−0.12	0.09	−0.04	0.52	−2.89	0.13	−2.17	0.12	0.35	0.09	0.27	0.14	−1.55	**< 0.01**	−0.02	0.93
Reminding × goal-setting	−0.09	0.29	−0.03	0.66	0.66	0.75	1.99	0.21	0.21	0.35	0.24	0.20	−0.97	0.10	−0.70	**0**.**01**
Role modeling × goal-setting	−0.24	**<0.01**	−0.08	0.18	−1.75	0.35	−1.60	**0.01**	0.26	0.21	0.20	0.28	−1.37	**0**.**01**	−0.05	0.86
Reminding × role modeling × goal-setting	0.32	**<0.01**	0.33	**<0.01**	3.68	0.07	3.94	**<0.01**	−0.58	**0**.**01**	−1.03	**<0.01**	−0.44	0.44	0.54	0.07

Significant *p*-values are in bold.

^a^ ITT, intention-to-treat.

^b^ PASE, Physical Activity Scale for the Elderly.

^c^ SEE, Self-Efficacy for Exercise Scale.

^d^ SCD, subjective cognitive decline.

^e^ MoCA, Montreal Cognitive Assessment.

^f^ Adjusted for age, sex, education, occupation, smoking, and baseline PASE.

^g^ Models adjusted for age, sex, education, occupation, smoking, and baseline ESS.

^h^ Adjusted for age, sex, education, occupation, smoking, and baseline SCD.

^i^ Adjusted for age, sex, education, occupation, smoking, and baseline MoCA.

* After Ln transformation; ×, interaction effects.

### 3.3 Cognitive function

The effects of reminding (*B* = −0.39, *p* = 0.02) and role modeling (*B* = −0.53, *p* = 0.01) on SCD were significant after adjusting for age, gender, education, occupation, smoking, and baseline cognitive function. The effect of role modeling (*B* = 0.68, *p* = 0.03) on MoCA was significant. In the interaction analysis, there was a synergistic effect between reminding, role modeling, and goal-setting (*B* = −1.03, *p* < 0.01). There was an antagonistic effect among the three implementation strategies (*B* = −0.70, *p* = 0.02) (see Table 3).

### 3.4 Self-efficacy

The effects of role modeling (*B* = 7.47, *p *< 0.01) and goal-setting (*B* = 4.39, *p* = 0.02) on self-efficacy were significant at 12 weeks. The effects of reminding (*B* = 0.61, *p* < 0.01), role modeling (*B* = 0.64, *p* < 0.01), and goal-setting (*B* = 0.60, *p* < 0.01) on self-efficacy were significant after adjusting for age, gender, education, occupation, smoking, and baseline self-efficacy at 12 weeks. In the interaction analysis, there was a synergistic effect among the three implementation strategies (*B* = 3.94, *p* < 0.01) (see Table 3).

### 3.5 Cost-effectiveness

The cost ranged from 93.41 to 148.49 RMB to improve one unit of PA, 4,487.18 to 5,833.33 RMB to enhance one unit of SCD, and 4,402.52 to 7,000.00 RMB to enhance one unit of overall cognitive function. Notably, role modeling exhibited the highest cost-effectiveness value compared with that of the other two implementation strategies (see Table 4).

**Table 4:** Cost-effectiveness analysis.

Implementation strategies	E11	E22	E33	C4	C/E1	C/E2	C/E3
Reminding	47.14	−1.4	1.1	7,000	148.49	5,000.00	6,363.64
Role modeling	74.94	−1.56	1.59	7,000	93.41	4,487.18	4,402.52
Goal-setting	62.53	−1.20	1.0	7,000	119.95	5,833.33	7,000.00

E1: Physical Activity Scale for the Elderly (PASE) difference.

E2: subjective cognitive function (SCD) difference.

E3: MoCA (Montreal Cognitive Assessment) difference.

C: total cost.

### 3.6 Sensitivity analysis

Sensitivity analyses were performed using a PP analysis. The effects of role modeling (*B* = 0.38, *p* < 0.01) and goal-setting (*B* = 0.28, *p* < 0.01) on PASE were significant at 12 weeks. The effects of reminding (*B* = 0.18, *p* = 0.02), role modeling (*B* = 0.01, *p* < 0.01), and goal-setting (*B* = 0.16, *p* = 0.02) on PASE were significant at 12 weeks after adjusting for age, gender, education, occupation, smoking, and baseline PA. Regarding other outcomes, the results from the PP analysis were similar to those of the primary analysis (see Supplementary Table S2).

## 4 Discussion

Our study found that the effects of the three implementation strategies, namely, reminding, role modeling, and goal-setting, in PA among older adults with MCI were significant. Role modeling was the best implementation strategy to facilitate the implementation of the core intervention. Ultimately, the core intervention (X-CircuiT and health education) and the implementation strategy (role modeling) were the most economical and effective intervention packages to improve PA level and cognitive function among people with MCI.

Role modeling could significantly increase PA levels in community-dwelling older adults with MCI. This was supported by findings in previous studies. For example, the REACT PA intervention using leaders with at least Register of Exercise Professionals—Level 3 detected significant differences in moderate- to vigorous-intensity PA and PASE (39). In addition, one multicenter RCT found that PA behavior change interventions involving leaders were effective in improving PA levels in patients with breast cancer (40). In qualitative studies, leaders were found to play a crucial role in promoting PA participation. They must lead by example and create and foster a culture and environment of mutual support in PA (41). The positive effects of role modeling on the PA level could be explained by the promotion of similar behaviors and emotional arousal of SCT (42). The International Society for Physical Activity and Health (ISPAH) advocacy document “Eight Investments That Work in Physical Activity” mentions the use of positive role models as a key facilitator to motivate and encourage population-level PA (43). Individuals could emulate competent and well-known role models and imitate their behaviors (42).

We found that role modeling had a positive effect on cognitive function, whereas reminding and goal-setting did not show a significant cognitive enhancement effect. Previous studies have shown that improving cognitive function through PA requires a specific duration [12 weeks (44)] and dosage [150 min per week of moderate intensity, 75 min of vigorous intensity, or an equivalent combination of both (45)] to improve cognitive function. The reason might be the target dosage could not be achieved due to chronic health conditions. In this study, although participants were asked to achieve the target intervention duration and dosage, some individuals reduced their PA levels due to physical limitations such as pain, hypertension, and pulmonary disease. The reason why role modeling improved cognitive function may be due to the emergence of social factors during the intervention (46). Social interaction in PA could reduce loneliness, increase enjoyment and effort, and get a sense of familiarity and humor, especially when exercising with the same age and functional ability (47). As a result, role modeling, as an implementation strategy for improving cognitive function, could be the preferred implementation strategy in further studies.

Implementation strategy of reminding could improve PA in older people with MCI. This was in line with previous studies. One study (48) designed a PA reminder device “Raya” that was designed to focus on humanization and empathy. This device could be customized to match the users' preferences, offering options such as puppies, cats, and turtles. The results showed that the majority of users reported that Raya increased their willingness to engage in PA (48). A previous study (15) evaluated the effect of receiving text messages in 12 weeks on adherence to home-based strengthening exercises among individuals aged 50 years and older, and the results showed that the group who received text messages exercised more than the control group (who only used booklets). The underlying mechanism might be improved self-efficacy and more encouragement through reminding (15). A meta-analysis (49) showed that sending exercise reminder messages promoted older adults to engage in moderate to vigorous PA and the effect persisted for up to 1 year. Additionally, both personalized and non-personalized reminders were found to be effective in promoting PA. The WHO (50) recommended using mobile technology to improve older adults' health due to its advantages such as instant transmission, low cost, and widespread availability. In addition, when using short messaging service (SMS) reminders, some factors, such as duration, type, tone, frequency, sequence, and one-way/bidirectional characteristics, should be considered (50). Overall, reminding is an economical, convenient, and universal intervention component that can promote PA in the MCI population, but careful attention should be paid to the method of sending messages.

Goal-setting has been identified as another effective implementation strategy for improving PA levels among individuals with MCI. This finding was in line with another study using a goal-setting intervention for PA in middle-aged adults, which was achieved by making 12 biweekly phone calls, which resulted in a significant increase in PA levels and self-perceived physical benefits (51). Another multicenter RCT study found that goal-setting was critical in the implementation of the cognitive rehabilitation program in 239 patients with mild to moderate dementia in the community (52). However, some studies did not conclude the effectiveness of goal-setting in improving the PA level. A systematic review of goal-setting in rehabilitation showed that goal-setting did not improve physical function among older adults (53). The possible reason might be that the study lacked a process evaluation and a clear description of the usual care, and lacked a variety of approaches to goal-setting of the included studies. It deserves attention that goals need to be specific, accepted, and provided with feedback and a deadline (54).

The results of the interaction analysis suggested that a single implementation strategy improved the PA level. It was also characterized by its simplicity and cost-effectiveness. The core intervention (X-CircuiT PA and health education) and the implementation strategy (role modeling) can be applied as a PA intervention package in community-dwelling older adults with MCI. The core points of the implementation strategy (role modeling) in this study are “leading” and “role model.” The person who performs the role modeling should have a certain level of prestige within the population and be willing to demonstrate the behavior. Therefore, people emulate competent and famous people, and through their leadership, they can learn correct and incorrect behaviors (42). Furthermore, role modeling characteristics are required for the leader-assisted intervention components. In the subsequent promotion and application of the MCI intervention package, understanding the potential mechanism of core intervention and implementation strategy can better debug the auxiliary intervention components and implement the core intervention effectively.

This study had some limitations. First, the core intervention of our study included the X-CircuiT plus education and the X-CircuiT was fixed, which did not consider the diversity of the PA context. Dynamic and diverse the PA content could be considered to maintain interest in the future. Second, three implementation strategies were selected based on SCT and qualitative interview results, but other effective implementation strategies may exist. Future research could suggest research-specific implementation strategies based on the preferred values of the study participants.

## 5 Conclusion

The study aimed to explore the most effective implementation strategy for improving PA and cognitive function using the MOST. This study presented the positive findings from a study of a 2 × 2 × 2 factorial randomized design, with the results showing that three implementation strategies (viz., reminding, role modeling, and goal-setting) could promote PA in community-dwelling older adults with MCI and that role modeling could additionally improve cognitive function. Robust synergy effects among the three implementation strategies have not been identified. Therefore, this study finally concluded that the core intervention (X-CircuiT PA and health education) and role modeling may be suitable as an intervention package characterized by applicability and cost-effectiveness to support clinicians in providing PA management for community-dwelling older adults with MCI.

## Data Availability

The raw data supporting the conclusions of this article will be made available by the authors, without undue reservation.
