# Supplementary material for: Optimizing the physical activity intervention for older adults with mild cognitive impairment: a factorial randomized trial
Source: Front Sports Act Living. 2024 May 7;6:1383325. doi: 10.3389/fspor.2024.1383325 (PMC11106430; doi:10.3389/fspor.2024.1383325)
Supplement: Supplementary file 1 [file Table1.docx]

Optimizing the physical activity intervention for older adults with mild cognitive impairment: A factorial randomized trial

**Zhanfang Shao, MD ^1,2^, Jundan Huang, PhD ^2^, Hui Feng, PhD ^2^, Mingyue Hu, PhD ^2^***

*** Correspondence:** Central South University, Xiangya School of Nursing, Changsha, Hunan, China. Tel.:86-166338060520. mingyue.hu@csu.edu.cn (MYH)

Supplementary Figure S1. The excerpt of X-CircuiT actions
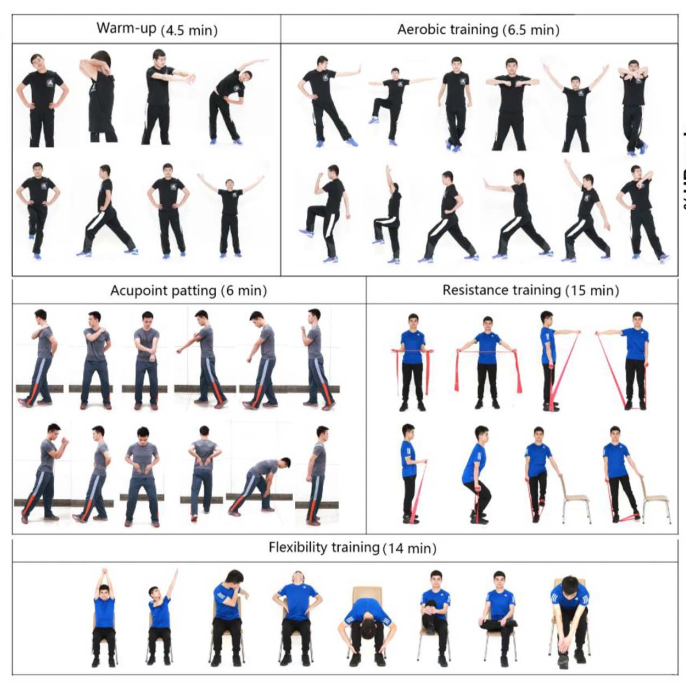


Supplementary Table S1. The cost of the intervention

| Intervention | cost |
| --- | --- |
| Labor cost | ¥ 3000/ per community (working in communities) |
| Core intervention |  |
| X-CircuiT | ¥ 0/person |
| Education | ¥ 3000/person |
| Implementation strategies |  |
| Role modeling | ¥ 0/person |
| Goal-setting | ¥ 0/person |
| Reminding | ¥ 0/person |
| Others | ¥ 1000 |
| Total | ¥ 7000/per community (about $1000/per community) |

Supplementary Table S2. Results of outcome (PP analysis)

| Implementation strategies | PASE ^1*^ | | | | ESS ^2^ | | | | SCD ^3^ | | | | MoCA ^4^ | | | | | |
| --- | --- | --- | --- | --- | --- | --- | --- | --- | --- | --- | --- | --- | --- | --- | --- | --- | --- | --- |
|  | No adjusted | | Adjusted ^5^ | | No adjusted | | Adjusted ^6^ | | No adjusted | | Adjusted ^7^ | | No adjusted | | | Adjusted ^8^ | | |
|  | B | *p* | B | *p* | B | p | B | p | B | p | B | p | | B | p | | B | p |
| Reminding | 0.19 | 0.25 | 0.18 | 0.02 | 2.82 | 0.18 | 0.61 | **< 0.01** | -0.37 | **0.08** | -0.41 | **0.04** | | -0.33 | 0.56 | | 0.91 | **< 0.01** |
| Role modeling | 0.38 | **< 0.01** | 0.01 | **< 0.01** | 7.35 | **< 0.01** | 0.64 | **< 0.01** | -0.62 | **< 0.01** | -0.56 | **0.01** | | 1.25 | **0.02** | | 0.89 | **< 0.01** |
| Goal-setting | 0.28 | **< 0.01** | 0.16 | **0.02** | 3.68 | 0.08 | 0.60 | **< 0.01** | -0.05 | 0.81 | -0.07 | 0.74 | | -0.08 | 0.88 | | 0.91 | **0.00** |
| Reminding × Role modeling | -0.10 | 0.19 | -0.04 | 0.53 | -2.90 | 0.15 | -1.89 | 0.21 | 0.32 | 0.13 | 0.26 | 0.17 | | -1.39 | **0.01** | | 0.00 | 0.99 |
| Reminding × Goal-setting | -0.06 | 0.45 | -0.02 | 0.76 | 0.65 | 0.77 | 2.48 | 0.14 | 0.20 | 0.38 | 0.21 | 0.30 | | -0.88 | 0.15 | | -0.67 | **0.02** |
| Role modeling × Goal-setting | -0.23 | **< 0.01** | -0.09 | 0.17 | -1.79 | 0.37 | -3.49 | **0.02** | 0.23 | 0.29 | 0.18 | 0.38 | | -0.01 | 0.97 | | -0.01 | 0.97 |
| Reminding × Role modeling × Goal-setting | 0.34 | **< 0.01** | 0.34 | **< 0.01** | 3.97 | 0.06 | 7.30 | **< 0.01** | -0.66 | **< 0.01** | -1.06 | **< 0.01** | | -0.22 | 0.70 | | 0.59 | **0.07** |

Notes: ^1^ PP: per-protocol; ^2^ PASE: physical activity scale for elderly; ^3^ ESS: Exercise self-efficacy scale; ^4^ SCD: Subjective cognitive decline; ^5^ MoCA: Montreal Cognitive Assessment; * After Ln transformation; ×: interaction effects; ^6^ Adjusted for age, sex, education, occupation, smoking, and baseline PASE; ^7^ Models Adjusted for age, sex, education, occupation, smoking, and baseline ESS; ^8^ Adjusted for age, sex, education, occupation, smoking, and baseline SCD; ^9^ Adjusted for age, sex, education, occupation, smoking, and baseline MoCA. Significant *p*-values are in bold.
